# Long noncoding RNAs as potential biomarkers and therapeutic targets in gallbladder cancer: a systematic review and meta-analysis

**DOI:** 10.1186/s12935-019-0891-1

**Published:** 2019-06-28

**Authors:** Yuan Zhong, Xiaochao Wu, Quanpeng Li, Xianxiu Ge, Fei Wang, Peiyao Wu, Xueting Deng, Lin Miao

**Affiliations:** Medical Center for Digestive Diseases, The Second Affiliated Hospital of Nanjing Medical University, Nanjing, 210011 China

**Keywords:** Gallbladder cancer, lncRNA, Prognosis, Meta-analysis

## Abstract

**Background:**

Mounting evidence has shown that long noncoding RNAs (lncRNAs) can play a substantial role in gallbladder cancer (GBC) development as tumor promotors or suppressors, and their abnormal expression is relevant to GBC patient outcomes. We completed this systematic review and meta-analysis to explore the clinical significance and mechanisms of lncRNAs in GBC.

**Methods:**

We conducted a comprehensive literature search and selected eligible records according to the inclusion and exclusion criteria. Hazard ratios (HRs) and odds ratios (ORs) were extracted or calculated to estimate the relationships of high lncRNA expression with GBC patient survival and clinical outcomes.

**Results:**

Eighteen studies were identified as eligible for this systematic review and meta-analysis. Heterogeneity among HRs of overall survival (OS) was notably high (I^2^ = 86.2%, p < 0.001). Subgroup analysis suggested that overexpression of lncRNAs in a group that is upregulated in GBC showed a significant association with poor OS (HR = 2.454, 95% CI 2.004–3.004, I^2^ = 0%). Conversely, overexpression of lncRNAs in a downregulated group was markedly related to good OS (HR = 0.371, 95% CI 0.267–0.517, I^2^ = 0%). High expression levels of lncRNA AFAP1-AS1, MALAT1 and ROR were positively correlated with tumor size. Expression of lncRNA LET, LINC00152 and HEGBC exhibited a positive correlation with high T status. LncRNA LINC00152, HEGBC, MALAT1 and ROR showed a marked correlation with positive lymph node metastasis (LNM), while lncRNA GCASPC, MEG3, LET and UCA1 had the opposite effect. High expression levels of lncRNA HEGBC, PAGBC, PVT1 and UCA1 predicted high tumor node metastasis (TNM) stages, while lncRNA LET, GCASPC and MEG3 indicated low TNM stages. We also summarized the mechanisms of lncRNAs in GBC.

**Conclusion:**

Aberrant expression of several lncRNAs was indicative of the prognosis of GBC patients, and lncRNAs showed promise as biomarkers and therapeutic targets for GBC.

## Background

Gallbladder cancer (GBC) is the most common malignant extrahepatic biliary duct tumor with high malignancy [1]. Statistics show an estimated 12,360 new gallbladder cancer and other biliary cancer cases and 3960 related deaths in the United States in 2019 [2]. Only when the tumor grows up to a large size do symptoms appear, such as pain, jaundice and fever [3]. Because of the asymptomatic characteristics in the early stage, most patients are diagnosed late and miss the best opportunity for surgical resection, which is the only possible curative treatment for GBC. The 5-year survival rates are 85.9% and 56.1% for T1 (tumor was localized at the mucosal or lamina propria) and T2 (tumor invaded the subserosal layer) tumors, but only 19.2% and 14.1% for T3 and T4 tumors (tumor invasion was over the serosa) [4]. Thus, exploring novel biomarkers and therapeutic targets for GBC is a top priority.

Long noncoding RNAs (lncRNAs) play vital roles in cell processes, such as cell proliferation, differentiation, DNA damage response, chromosomal imprinting, etc. via transcriptional, posttranscriptional or epigenetic regulation of gene expression, rather than functioning as coding RNAs [5, 6]. Abnormal lncRNA expression is tightly correlated with tumorigenesis and progression, and lncRNAs act as not only tumor promotors but also suppressors. For example, Di Huang et al. [7] found that NF-κB-interacting lncRNA (NKILA) increased T cell sensitivity to activation-induced cell death (AICD) by inhibiting NF-κB, which promoted immune evasion in breast cancer. A p53-regulated lncRNA (loc285194), a tumor suppressor, could inhibit colon cancer cell growth and proliferation by repressing miR-211 [8].

Wu et al. [9] demonstrated that in gallbladder cancer, metastasis-associated lung adenocarcinoma transcript 1 (MALAT1) promoted GBC cell proliferation and metastasis through the activation of the ERK/MAPK pathway. In the following years, substantial numbers of lncRNAs related to GBC development and patient outcomes were discovered, such as LET [10], GCASPC [11], ANRIL [12], etc. The results of these studies have not been systematically reviewed and analyzed. Thus, we thoroughly collected all relevant studies and summarized the results to explore the potential prognostic value of lncRNAs in GBC.

## Materials and methods

### Literature search

A systematic literature search was conducted by two authors (Yuan Zhong and Xiaochao Wu) through PubMed, Web of Science, Embase and the Cochrane Library (up to March 21, 2019). The following search terms were used with Boolean conjunctions: (“lncRNA” OR “lincRNA” OR “long noncoding RNA” OR “long untranslated RNA” OR “long intergenic noncoding RNA”) AND (“gallbladder cancer” OR “gallbladder carcinoma” OR “carcinoma of gallbladder” OR “gallbladder tumor” OR “cancer of gallbladder” OR “gallbladder neoplasm”).

### Selection criteria

Studies from the literature search were included based on the following criteria: (1) LncRNA expression was detected in gallbladder cancer tissues; (2) the diagnosis of gallbladder cancer was confirmed by pathology and histology; (3) the relationship between lncRNA expression and gallbladder cancer patient prognosis was investigated, including survival and clinicopathological parameters; and (4) sufficient data were provided for the hazard ratio (HR) and the 95% confidence interval (95% CI) of survival or the odds ratio (OR) and the 95% CI of clinicopathological parameters. Meanwhile, unsuited studies were excluded by the following criteria: (1) animal studies, reviews, editorials and letters; (2) duplicate records; and (3) studies without sufficient data.

### Data extraction and quality assessment

Two investigators extracted requisite information from each eligible study: first authors, publication year, country, lncRNA type, sample size (high/low), sample type, lncRNA detection method, the HR and 95% CI of lncRNA for survival and patient number for histological grade, TNM stage, LNM, etc. Engauge Digitizer v.10.11 software and Tierney’s spreadsheet were utilized to obtain the HR when it was not reported directly [13]. The HR from multivariate analysis was preferred over that from the univariate analysis because the multivariate analysis considered the influence of confounding factors. The quality assessment of each eligible study was performed in accordance with the Newcastle–Ottawa Quality Assessment Scale (NOS) [14].

### Statistical analysis

As different lncRNAs could act inversely in GBC, pooling HRs and ORs directly would lead to great heterogeneity. Thus, we assessed the heterogeneity among studies and conducted the subgroup analysis of HRs based on the role of the lncRNA in gallbladder cancer. The heterogeneity of the results was estimated by the Q test and I^2^ statistics. When I^2^ ≥ 50%, the random pooling model was selected. In contrast, the fixed pooling model was used. HR > 1 suggested a significant association of lncRNA overexpression with poor survival, and HR < 1 indicated that high lncRNA expression predicted long survival [15]. All these calculations were completed with the assistance of STATA v.15 software.

## Results

### Characteristics of the included studies

**Fig. 1:**
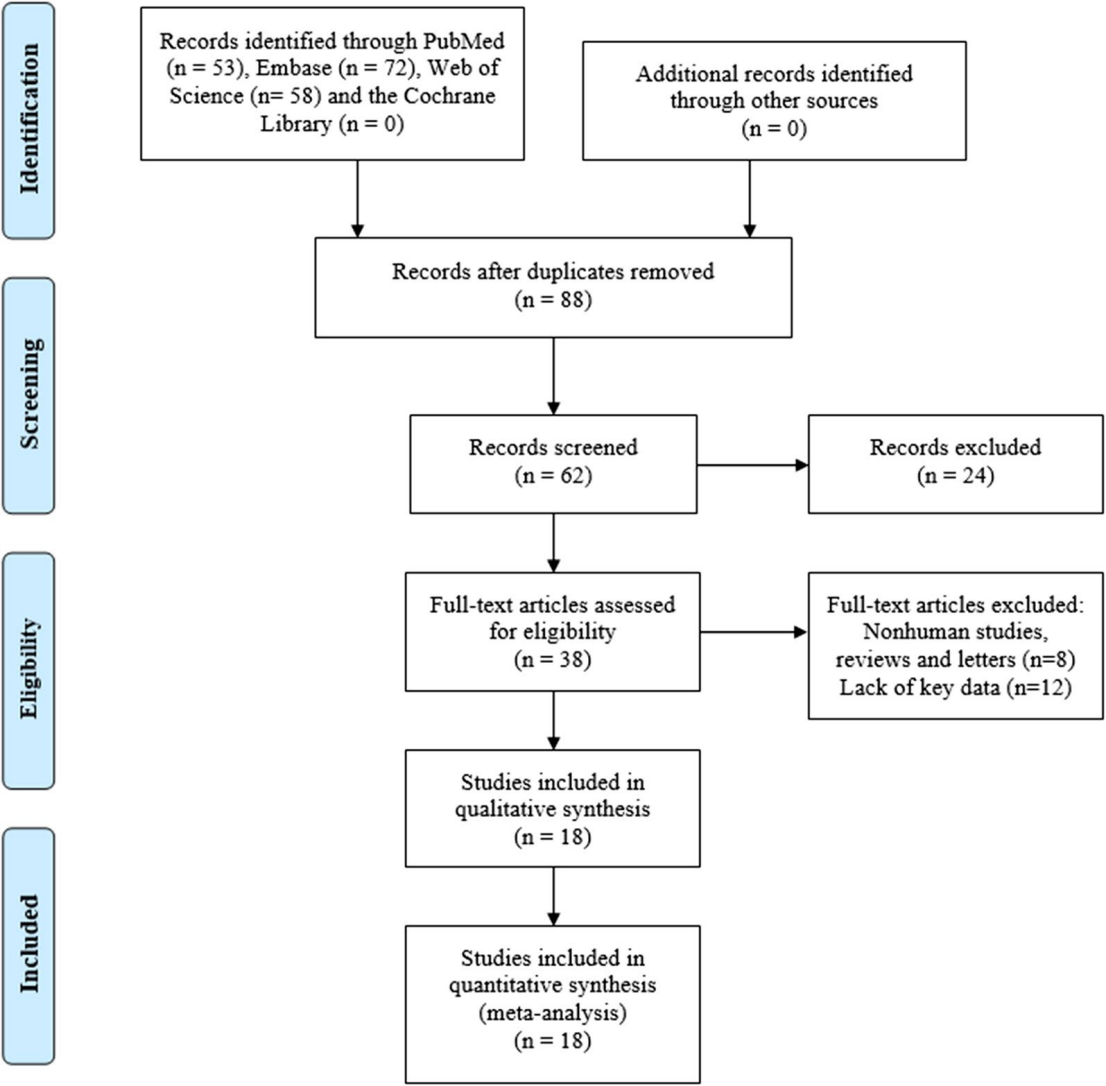
Flow diagram of this systematic review and meta-analysis

As shown in Fig. 1, a total of 18 articles published between 2015 and 2019 with 1001 gallbladder patients were included in our analysis. The 15 relevant lncRNAs were as follows: LET [10], GCASPC [11], LINC00152 [16, 17], MEG3 [12, 18], ANRIL [12], MALAT1 [19], MINCR [20], AFAP1-AS1 [21], UCA1 [22], PAGBC [23], Loc344887 [24], ROR [25], HEGBC [26], H19 [27, 28] and PVT1 [29]. The 12 studies included one study of 702 gallbladder cancer patients that used OS to estimate patient survival and 3 studies of 217 patients that used both OS and disease-free survival (DFS). All lncRNA expression levels were detected by quantitative real-time polymerase chain reaction (qRT-PCR). All studies were conducted in China and all the samples were tissues. Furthermore, 4 studies reported HRs directly. More details are shown in Table 1.

**Table 1:** Characteristics of the included studies

First author	Year	LncRNA	Expression	Sample size (high/low)	Cut-off value	PT	Survival	Survival analysis	HR availability	NOS score
Mingzhe Ma	2015	LET	Downregulated	128 (47/81)	Median	No	OS, DFS	U, M	Text	8
Mingzhe Ma	2016	GCASPC	Downregulated	89 (45/44)	Median	No	OS, DFS	U, M	K–M curve	8
Qiang Cai	2016	LINC00152	Upregulated	40 (23/17)	Fold change	No	NA	NA	NA	6
Bo Liu	2016	MEG3	Downregulated	84 (42/42)	Median	No	OS	U	K–M curve	7
		ANRIL	Upregulated	84 (42/42)	Median	No	OS	U	K–M curve	
Shouhua Wang	2016	MALAT1	Upregulated	30 (15/15)	Median	No	OS	U	K–M curve	8
Shouhua Wang	2016	MINCR	Upregulated	35 (18/17)	Median	No	OS	U	K–M curve	8
Fei Ma	2017	AFAP1-AS1	Upregulated	40 (19/21)	Mean	No	OS	U	K–M curve	6
Qiang Cai	2017	LINC00152	Upregulated	35 (18/17)	Median	No	OS	U	K–M curve	6
Qiang Cai	2017	UCA1	Upregulated	45 (23/22)	Median	No	OS	U	K–M curve	8
Xiangsong Wu	2017	PAGBC	Upregulated	77 (38/39)	Median	No	OS	U	Text	8
Xiaocai Wu	2017	Loc344887	Upregulated	22 (15/7)	NA	No	NA	NA	NA	5
Shouhua Wang	2017	ROR	Upregulated	30 (14/16)	Median	No	OS	U	K–M curve	7
Longyang Jin	2018	MEG3	Downregulated	50 (24/26)	Mean	No	OS	U, M	Text	8
Liang Yang	2018	HEGBC	Upregulated	102 (51/51)	Median	No	OS	U	K–M curve	8
Shouhua Wang	2018	H19	Upregulated	20 (12/8)	NA	No	NA	NA	NA	6
Shouhua Wang	2019	H19	Upregulated	24 (13/11)	Median	No	OS	U	K–M curve	6
Jianan Chen	2019	PVT1	Upregulated	66 (33/33)	NA	NA	OS	U, M	Text	8

U, univariate analysis; M, multivariate analysis; PT, preoperative treatment; K–M curve, Kaplan–Meier curve; NA, not available

### The relationship between lncRNAs and patient survival

**Fig. 2:**
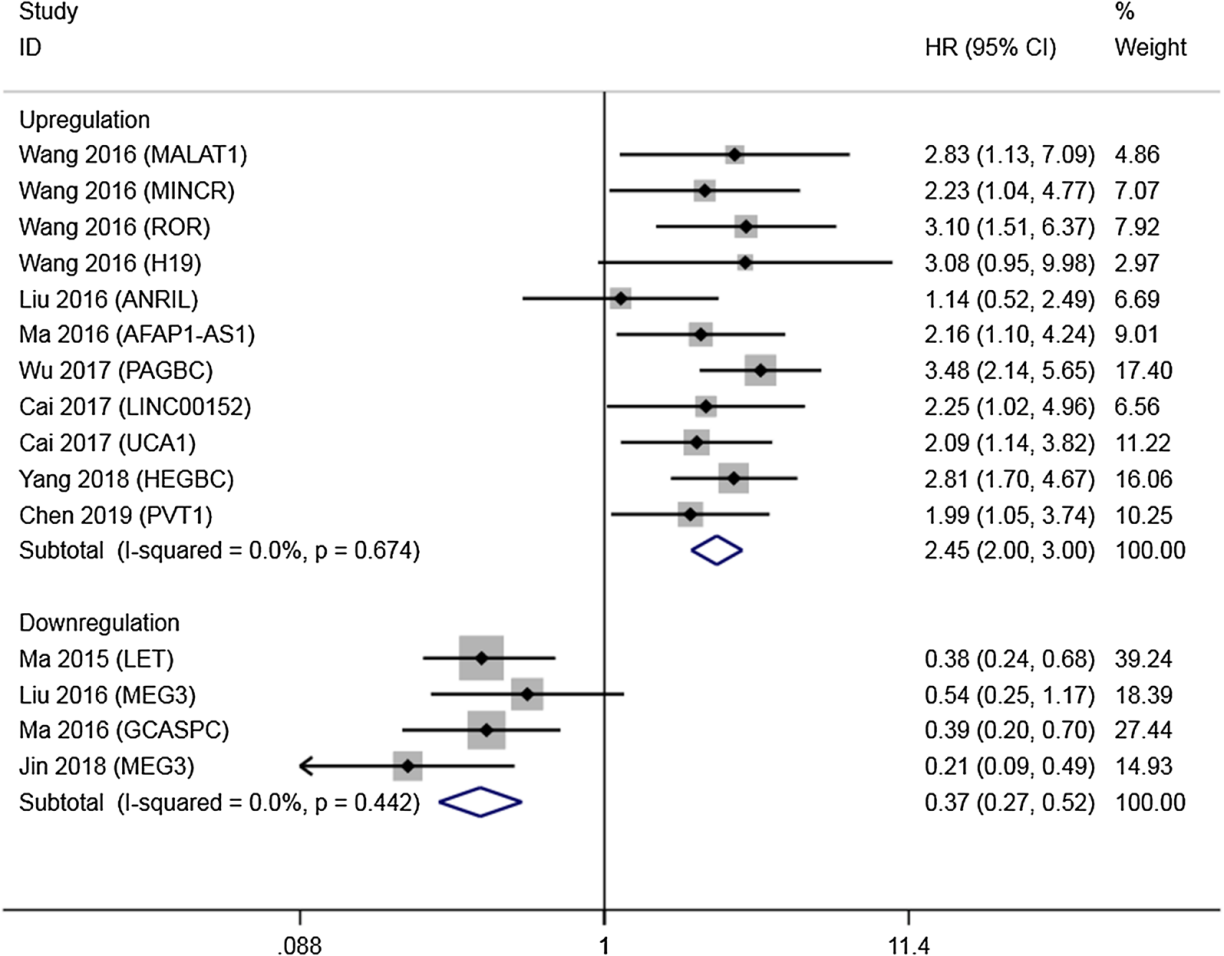
Subgroup analysis of OS by lncRNA expression in GBC tissues

**Fig. 3:**
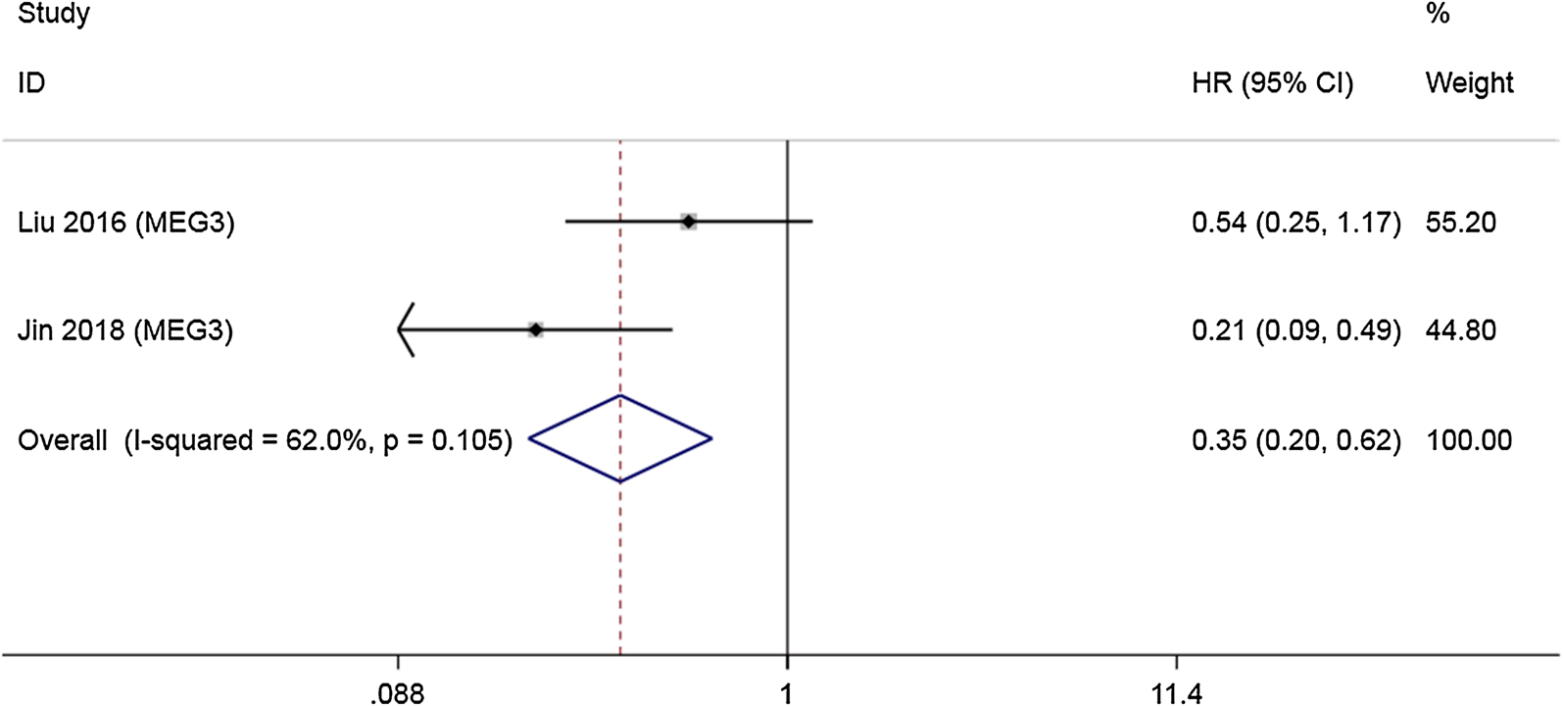
Forest plot of HRs of high MEG3 expression and DFS

**Fig. 4:**
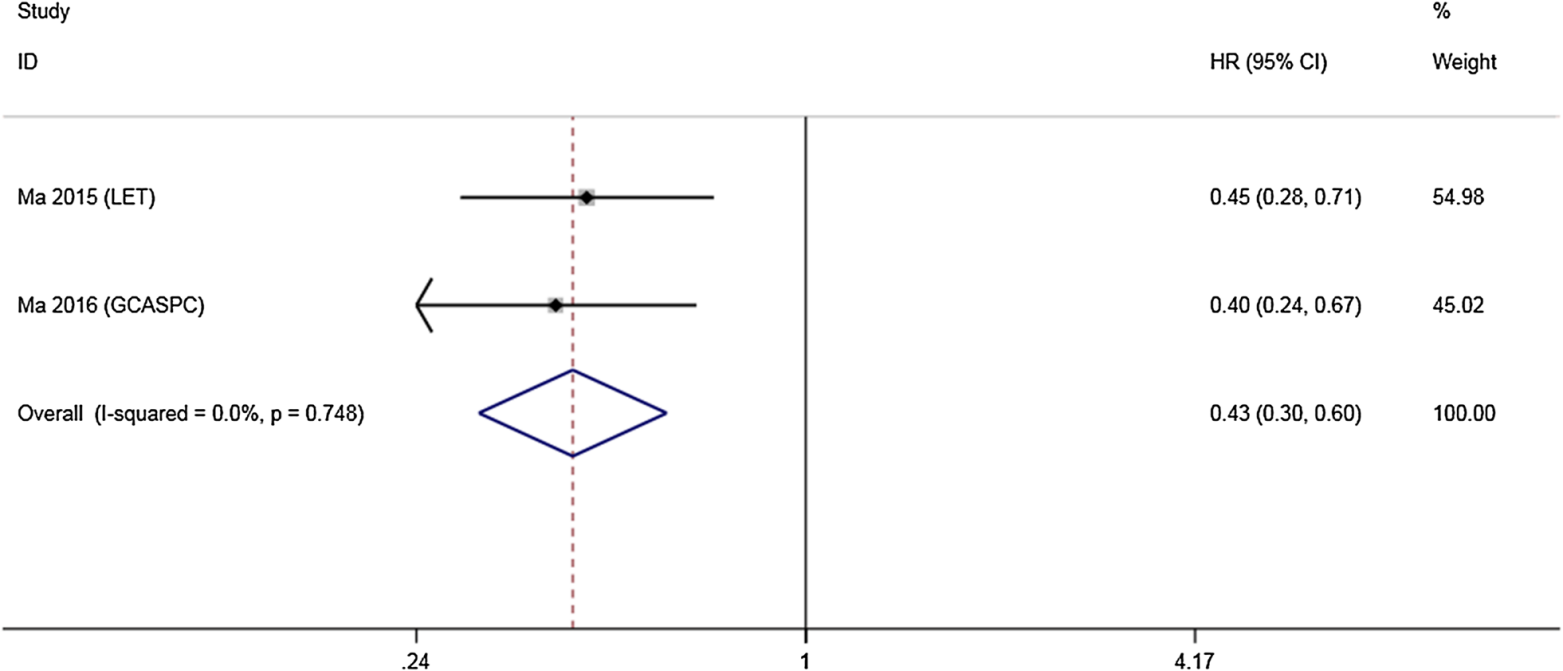
Forest plot of HRs of high lncRNA expression and DFS

LncRNAs LET, GCASPC and MEG3 were downregulated in gallbladder cancer tissues and acted as cancer suppressors, while the remaining 12 lncRNAs (LINC00152, ANRIL, MALAT1, MINCR, AFAP1-AS1, UCA1, PAGBC, Loc344887, ROR, HEGBC, H19, PVT1), were upregulated in cancer tissues and promoted gallbladder cancer progression. Heterogeneity among HRs of OS was markedly high (I^2^ = 86.2%, p < 0.001; Fig. 2). The subgroup analysis suggested that high expression levels of lncRNAs in the upregulation subgroup were significantly related to poor OS (pooled HR = 2.454, 95% CI 2.004–3.004, I^2^ = 0%). In contrast, increased levels of LET, MEG3 (Fig. 3) and GCASPC were favorable factors in OS (pooled HR = 0.371, 95% CI 0.267–0.517, I^2^ = 0%; Fig. 2). We also found that high expression levels of LET and GCASPC were markedly associated with DFS (pooled HR = 0.426, 95% CI 0.302–0.601; Fig. 4).

### The relationship between lncRNAs and clinicopathological outcomes

**Fig. 5:**
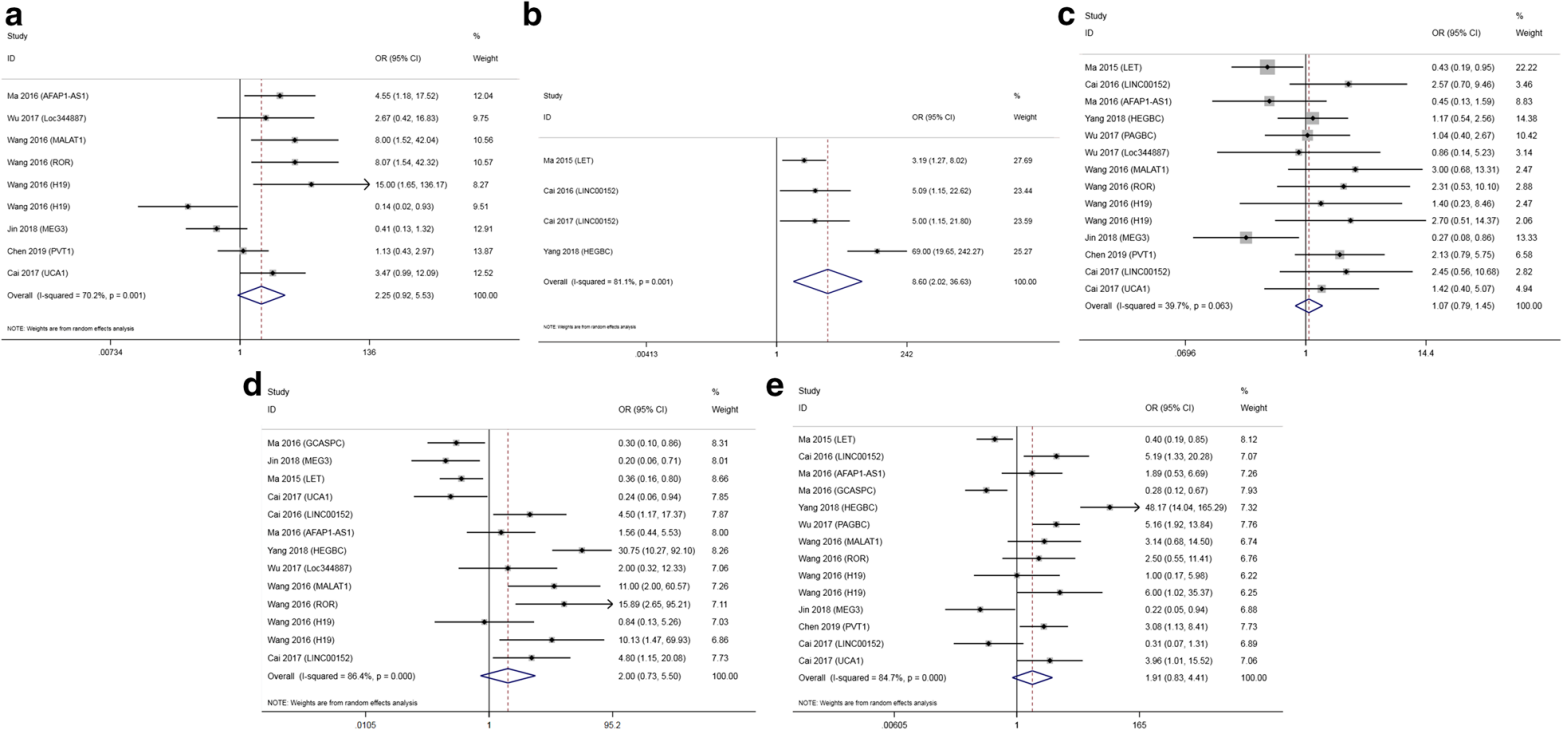
Forest plots of the association of high lncRNA expression with clinicopathological parameters: **a** tumor size; **b** T status; **c** histological grade; **d** LNM; and **e** TNM stage

The results indicated that increased expression of AFAP1-AS1, MALAT1 and ROR was significantly correlated with large tumor size, and overexpression of LET, LINC00152 and HEGBC indicated advanced T status. LET and MEG3 showed a positive association with better histological grade. Moreover, LINC00152, HEGBC, MALAT1 and ROR exhibited a notable correlation with positive LNM (LINC00152: OR = 4.500, 95% CI 1.166–17.373; HEGBC: OR = 30.750, 95% CI 10.266–92.103; MALAT1: OR = 11.000, 95% CI 1.998–60.572; ROR: OR = 15.889, 95% CI 2.652–95.208). In contrast, GCASPC, MEG3, LET and UCA1 were favorable factors for LNM (GCASPC: OR = 0.297, 95% CI 0.103–0.860; MEG3: OR = 0.201, 95% CI 0.057–0.713; LET: OR = 0.360, 95% CI 0.162–0.802; UCA1: OR = 0.242, 95% CI 0.062–0.942). Four upregulated lncRNAs (HEGBC, PAGBC, PVT1, UCA1) were unfavorable factors for TNM stage, while three lncRNAs (LET, GCASPC, MEG3) showed an opposite trend. All lncRNAs had no remarkable relationship with age or sex (age: p = 0.146 and sex: p = 0.848). More details are shown in Fig. 5.

**Fig. 6:**
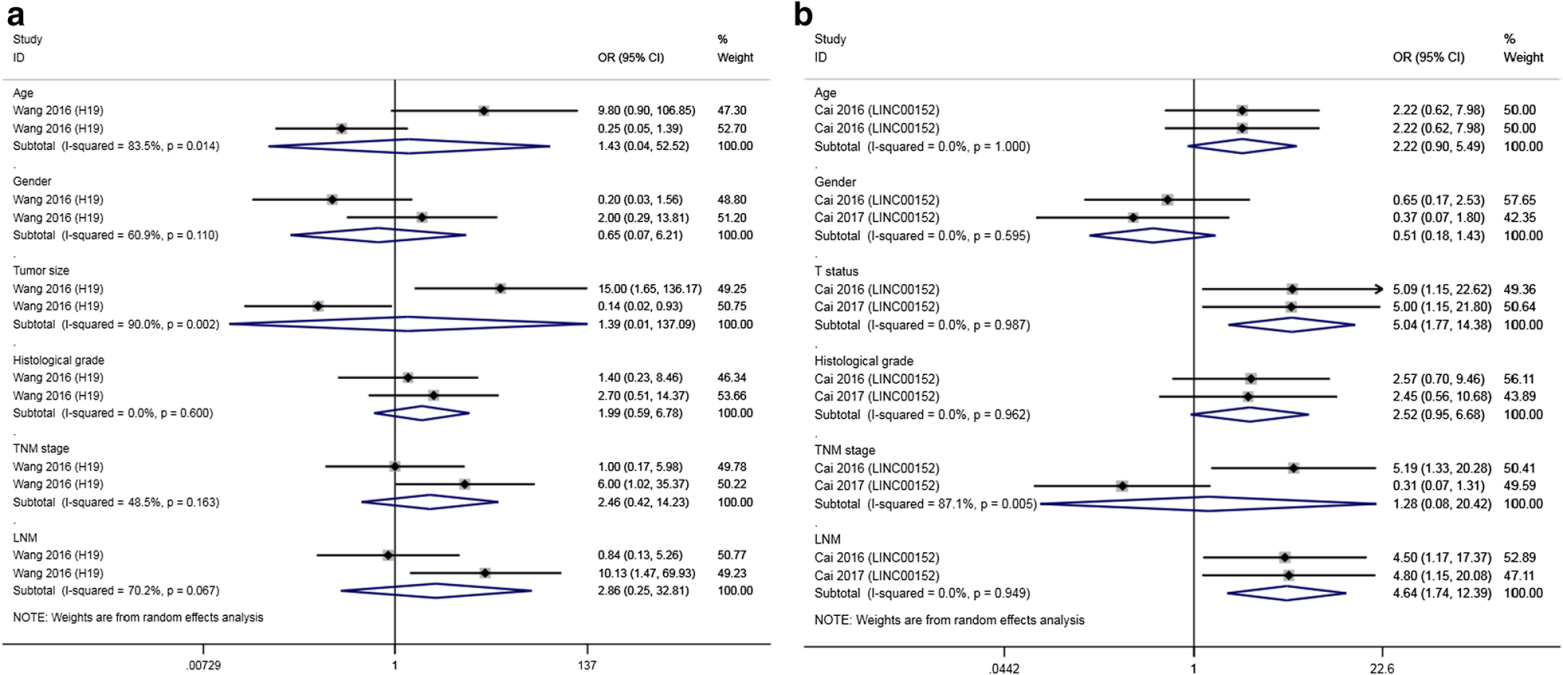
Forest plots of the association of two lncRNAs with clinicopathological parameters: **a** H19; **b** LINC00152

Both LINC00152 and H19 were explored by two studies on their association with clinicopathological features. As presented in Fig. 6, no obvious relevance of H19 to clinical parameters was found. LINC00152 overexpression was remarkably related to advanced T status (OR = 5.045, 95% CI 1.769–14.384) and positive LNM (OR = 4.639, 95% CI 1.737–12.390) but not TNM stage (p = 0.862).

### Publication bias and sensitivity analysis

**Fig. 7:**
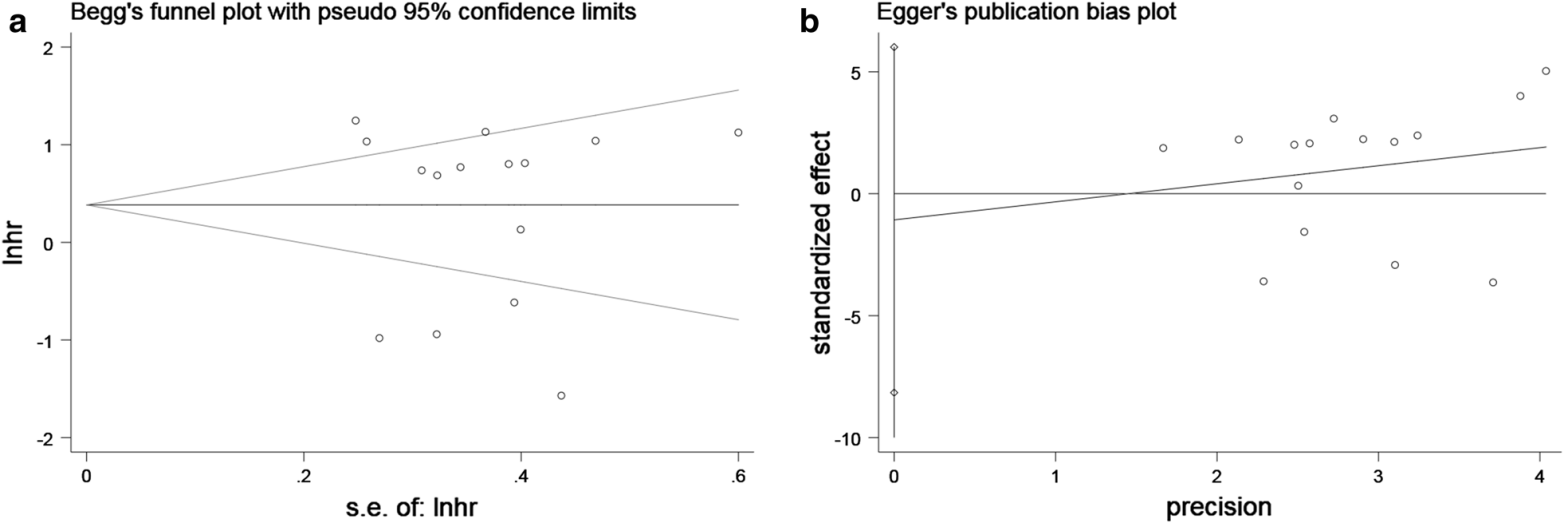
Tests for publication bias of OS: **a** Begg’s test; **b** Egger’s test

**Fig. 8:**
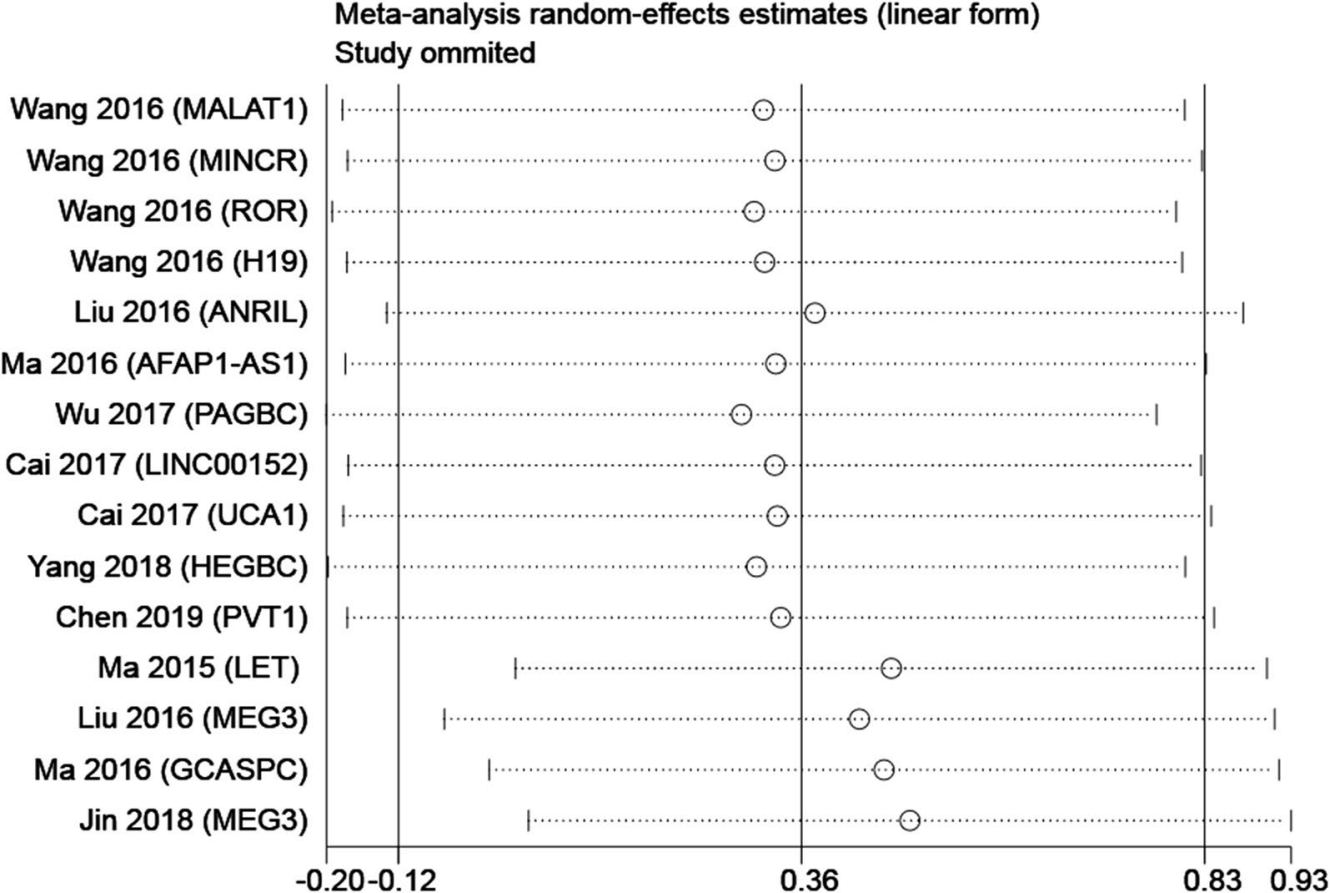
Sensitivity analysis of studies on OS

Egger’s publication bias plot and Begg’s funnel plot were constructed to explore the potential publication bias. Two plots did not display apparent asymmetry (Egger’s test: p = 0.749 and Begg’s test: p = 0.553), which showed no significant publication bias (Fig. 7). In addition, the sensitivity analysis suggested the robustness of the results because eliminating any study did not change the results significantly (Fig. 8).

### Action mechanisms of lncRNAs in gallbladder cancer

In addition, we concentrated on potential targets and pathways of the included lncRNAs in gallbladder cancer, as presented in Table 2. MEG3 and UCA1 could both target Enhancer of Zeste Homolog 2 (EZH2), despite their differential roles in gallbladder cancer. Furthermore, MINCR could stimulate EZH2 expression via targeting miR-26a-5p. MEG3 and ANRIL acted inversely on p53 and CyclinD1, which was consistent with their roles in GBC.

**Table 2:** Summary of lncRNAs with their potential targets and pathways

LncRNA type	Expression	Potential target(s)	Pathways	References
LET	Downregulated	NA	↓Cell proliferation, invasion and tumor growth; ↑apoptosis; G0/G1 arrest	[10]
LINC00152	Upregulated	miR-138	↑Cell proliferation, migration, invasion and EMT; ↑tumor peritoneal spreading and metastasis; ↓apoptosis; PI3K/AKT pathway; LINC00152/miR-138/HIF-1α pathway	[16, 17]
MEG3	Downregulated	EZH2, LATS2	↓Cell proliferation, invasion, EMT and tumor growth; ↑cell apoptosis; G1/G0 arrest; ↑EZH2 ubiquitination; ↑p53; ↓CDK4 and CyclinD1	[12, 18]
ANRIL	Upregulated	NA	↑Cell proliferation and tumor growth; ↓apoptosis; ↓p53; ↑CyclinD1	[12]
GCASPC	Downregulated	Pyruvate carboxylase	↓Cell proliferation and tumor growth; ↓pyruvate carboxylase	[11]
MALAT1	Upregulated	miR-206	↑Cell proliferation and invasion; ↓cell apoptosis; ↓miR-206; ↑KRAS and annexin a2	[9, 19, 32, 33]
MINCR	Upregulated	miR-26a-5p	↑Cell proliferation, EMT and invasion; ↓cell apoptosis; MINCR/miR-26a-5p/EZH2 axis	[20]
AFAP1-AS1	Upregulated	NA	↑Cell proliferation, EMT and tumor invasion	[21]
UCA1	Upregulated	EZH2	↑Cell proliferation, EMT and metastasis; ↓p21 and E-cadherin	[22]
PAGBC	Upregulated	miR-133b, miR-511	↑Cell proliferation, migration and invasion; ↑tumor growth and metastasis; ↑SOX4 and PIK3R3; AKT/mTOR pathway; PABPC1	[23]
Loc344887	Upregulated	NA	↑Cell proliferation, mobility, EMT and invasion	[24]
ROR	Upregulated	NA	↑Cell proliferation, migration, EMT and invasion	[25]
HEGBC	Upregulated	IL-11	↑Cell proliferation, migration; ↑tumor growth and metastasis; IL-11/STAT3 pathway	[26]
H19	Upregulated	AKT2	↑Cell proliferation and invasion; H19/miR-194-5p/AKT2 axis	[27, 28]
PVT1	Upregulated	miR-143	↑Cell proliferation, migration and invasion; ↑tumor growth; ↑MMP-2 and MMP-9; PVT1/miR-143/HK2 axis	[29]

↑, promote; ↓, inhibit

## Discussion

Gallbladder cancer is characterized by a difficult early diagnosis, strong invasion and poor prognosis. Although complete resection can eliminate gallbladder tumors confined to the mucosa, the majority of patients are diagnosed at a late stage, which means that tumors have disseminated to nearby organs or lymph nodes or distant body sites. Patients with advanced GBC lose their opportunity for curative radical cholecystectomy, while radiotherapy or chemotherapy tend to be limited and palliative because of their low sensitivity. Thus, investigations for novel biomarkers and therapeutic targets for GBC make sense practically. Noncoding RNAs including lncRNA, microRNA and circRNA play vital roles in GBC tumorigenesis and progression [30, 31]. Increased studies have reported the relationship between aberrant lncRNA expression and GBC, but the results have not yet been systematically evaluated or analyzed. Therefore, we aimed to summarize and analyze the results to obtain more accurate conclusions.

In this systematic review and meta-analysis, we explored the role of lncRNAs in patient survival and clinicopathological parameters. The heterogeneity among HRs of OS was notably high, possibly because of differential expression of lncRNAs in GBC tissues according to nearly non-existent heterogeneity in both subgroups. Overexpression of GBC promoters (MALAT1, MINCR, ROR, AFAP1-AS1, PAGBC, LINC00152, UCA1, HEGBC and PVT1) predicted poor OS, while high expression of GBC suppressors (LET, GCASPC and MEG3) indicated favorable OS. In addition, the expression levels of LET and GCASPC were positively related to long DFS.

With regard to clinicopathological outcomes, high expression of AFAP1-AS1, MALAT1 and ROR corresponded to a large tumor size. High expression of LET, LINC00152 and HEGBC predicted high T status. LET and MEG3 acted as indicators for low histological grade, while other lncRNAs showed no significance. LINC00152, HEGBC, MALAT1 and ROR functioned as markers for positive LNM, while GCASPC, MEG3, LET and UCA1 indicated negative LNM. In consideration of the tumor-promoting effects of UCA1, more studies with a large sample size need to be conducted to verify the conclusion. HEGBC, PAGBC, PVT1 and UCA1 predicted high TNM stage, and LET, GCASPC and MEG3 predicted low TNM stage. H19 showed no association with any parameter according to two studies with a small sample size. The overexpression of LINC00152 predicted high T status and positive LNM.

We also focused on the mechanisms of lncRNAs in GBC, which might be helpful for further studies. LINC00152 could promote GBC development via both the PI3K/AKT pathway and the LINC00152/miR-138/HIF-1α pathway. MEG3 could suppress GBC proliferation and metastasis through targeting both EZH2 and Large Tumor Suppressor 2 (LATS2). PAGBC plays an oncogenic role in GBC by targeting miR-133b and miR-511 and activating AKT/mTOR pathway.

Limitations need to be mentioned in this systematic review and meta-analysis. First, because all the included studies were from China, the conclusions might not be applicable to other populations. Second, estimates that exaggerate the magnitude of the effect might arise from the small sample size of most studies. Third, 11 HRs of OS were calculated from the K–M curves rather than being reported directly.

## Conclusions

Our study first systematically reviewed and estimated the association of abnormal lncRNA expression with GBC patient survival and clinical outcomes. We confirmed several lncRNAs, whether GBC promoters or GBC suppressors, as potential biomarkers and therapeutic targets for GBC. In consideration of the limitations of this study, more large-scale and high-quality studies on various ethnic populations are needed to form better conclusions of the value of lncRNAs in GBC.

## Data Availability

All data are included in this article.

## Abbreviations

lncRNA: long noncoding RNA
GBC: gallbladder cancer
HR: hazard ratio
OR: odds ratio
OS: overall survival
DFS: disease-free survival
95% CI: 95% confidence interval
qRT-PCR: quantitative real-time polymerase chain reaction
MALAT1: metastasis-associated lung adenocarcinoma transcript 1
LNM: lymph node metastasis
NOS: the Newcastle–Ottawa Quality Assessment Scale
MEG3: maternally expressed gene 3
ANRIL: antisense non-coding RNA at the INK4 locus
AFAP1-AS1: actin filament-associated protein 1 antisense RNA 1
PVT1: plasmacytoma variant translocation 1
HEGBC: high expressed in gallbladder cancer
LET: low expression in tumor
GSASPC: gallbladder cancer-associated suppressor of pyruvate carboxylase lncRNA
UCA1: urothelial carcinoma associated 1
MINCR: MYC-induced long noncoding RNA
ROR: regulator of reprogramming
PAGBC: prognosis-associated gallbladder cancer
LATS2: large tumor suppressor 2
AKT2: v-AKT murine thymoma viral oncogene homologue 2
